# Do lower extremity kinematics and training variables affect the development of overuse injuries in runners? - a prospective study

**DOI:** 10.1186/1757-1146-5-S1-O46

**Published:** 2012-04-10

**Authors:** Tobias Hein, Pia Janssen, Ursula Wagner-Fritz, Stefan Grau

**Affiliations:** 1 Department of Sports Medicine, Medical Clinic, University of Tübingen, Tübingen, Germany

## Background

The incidence of running injuries appears to be multi-factorial, e.g. with regard to training errors or kinematics of the lower extremity [1,2]. To date, studies examining differences between healthy and injured runners are mainly retrospective, and therefore not able to determine whether these differences are the cause or effect of injury. The goal of this prospective study is to evaluate whether the development of overuse injuries in initially healthy subjects is caused by alterations in lower extremity kinematics and/or training habits.

## Materials and methods

Since April 2009, 218 healthy runners (m=139, w=79) have been included in the study. Besides a standardized clinical examination, 3D-kinematics of both lower extremities were recorded according to ISB-recommendations [3]. Hip, knee and ankle joint motions were quantified by calculating Cardan angles [4]. Mean angular displacements and discrete parameters were calculated from 10 valid trials of barefoot running. All subjects were asked to complete and return weekly training logs with information about their weekly mileage, running time, and type of training sessions, as well as information about the occurrence of pain during training. Study duration lasted a maximum of 12 months or until diagnosis of an overuse injury.

## Results

Currently, 35 from 104 subjects who have completed the study have developed overuse injuries. Symptomatic runners ran more training kilometers per week than healthy runners, and showed a shift from slow and medium to fast endurance training sessions. Further, runners who generated overuse injuries exhibited differences in rear foot, ankle and knee kinematics compared to runners who remained uninjured during their participation.

## Conclusions

The initial results indicate that lower leg kinematics and training parameters are risk factors and consequently possible causes for developing overuse injuries. Further subjects will be included in the evaluation to enable a division into larger injury-specific groups.
